# Experimental Examination of Electrical Characteristics for Portland Cement Mortar Frost Damage Evaluation

**DOI:** 10.3390/ma13051258

**Published:** 2020-03-10

**Authors:** Yi Wang, Tamon Ueda, Fuyuan Gong, Dawei Zhang, Zhao Wang

**Affiliations:** 1 College of Civil Engineering and Architecture, Zhejiang University, Hangzhou 310058, China; wangyi@iis.u-tokyo.ac.jp (Y.W.); gongfy@zju.edu.cn (F.G.); 2 School of Civil and Transportation Engineering, Guangdong University of Technology, Guangzhou 510006, China; 3 School of Civil and Transportation Engineering, Shenzhen University, Shenzhen 518060, China; 4 Institute of Urban Innovation, Yokohama National University, Yokohama 240-8501, Japan; wangzhaoousyo@gmail.com

**Keywords:** frost damage, mortar, sodium chloride, electrical measurements, elastic modulus

## Abstract

Electrical measurements are promising for evaluation of frost damage of concrete, but the index is still controversial. In this paper, to propose an efficient index, various electrical characteristics were examined to correlate them with the mechanical property degradation of meso-scale mortar samples due to combined effects of sodium chloride and freeze–thaw cycles (FTCs). While the electrical responses of specimens were measured during FTCs, the mechanical properties were obtained from three-point bending tests after FTCs. Typical microstructural change after the damage was also analyzed by using a water absorption test. The results showed that no clear degradation tendency was observed for electrical resistivity at the lowest temperature, the activation energy or the freezing/thawing point change with the FTCs. The reduction in electrical resistivity at reference temperature has a consistent tendency with that of elastic modulus and flexural strength, thus can be an efficient index for quantitative frost damage evaluation. The change due to salt-frost damage is mainly due to the increase of connectivity rather than porosity.

## 1. Introduction

Concrete structures, such as bridges, roadways and walkways, are fragile to freeze–thaw cycles (FTCs) [1]. In winter, to protect the safety of vehicles and pedestrians, ice on the surface of concrete will be removed by spraying de-icing agents (e.g., sodium chloride (NaCl)). The de-icing agents can accelerate the frost damage of concrete; such damage has raised much concern in recent years [2]. Analytical studies have been conducted to predict the frost damage of concrete [3,4,5,6], but some discrepancies remain due to the complex of damage phenomena. In addition to salt scaling, which spoils the appearance of the concrete, more importantly, internal frost damage threatens safety. The frost damage may not only be induced by ice formation, but also contributed by the formation of Friedel’s salt. Thus, it is necessary to investigate the frost resistance of concrete combined with salt ingress [2,7,8]. Based on the acoustic emission results, Farnam et al. [9] found that the most severe frost damage did not occur at approximately 3 wt.% of NaCl solution but at 15 wt.% due to the salt crystallization, which reveals that the damage process is much more complicated than that explained by the existing models [5]. However, from the deformation measurement [10], in the case of 15 wt.% NaCl saturated sample under sealed condition, although the damage could occur due to the salt crystallization, no clear expansion with FTCs was observed. It means that different frost damage testing methods may lead to different results. Furthermore, our previous closed freeze–thaw test results showed that the strength and stiffness of NaCl solution-saturated mortar degrade with FTCs [11]. Therefore, to understand the damage mechanism, in addition to acoustic emission and strain, an alternative measurement to evaluate the damage of concrete is required.

Recently, investigating the frost damage of concrete via electrical measurement was considered as a great potential methodology because it is non-destructive and rapid data acquisition [12,13]. This technique has been applied to study concrete for decades [14], including the chloride diffusivity [15,16], damage detection during tensile loads [17,18], water penetration in concrete [19,20] and concrete internal moisture content [21]. It is believed that the electrical resistivity of concrete is related to the pore characteristics, saturation degree, temperature and pore solution compositions [22]. The relationship between the electrical conductivity of concrete and the porosity can be expressed as modified Archie’s law [23]. Moreover, the moisture effect to electrical resistivity can be quantified by Archie’s law [22,24], and the temperature effect can be described by the Arrhenius equation and the activation energy is the index for evaluation [25,26,27,28]. In addition, the electrical conductivity of cement paste can be estimated based on the pore solution ionic strength [29]. By analyzing the electrical response, the chloride ingress can be quantified for lifecycle prediction [30], which has been adopted in the AASHTO PP 84-19 [31] provisional specification for practical application. Therefore, the electrical properties change of concrete could be used as indicator for durability [32]. However, in actual application, how the combined effects of these parameters influence the electrical conductivity of concrete remains unclear.

Because ice formation is essential for understanding the frost damage of concrete [33], electrical measurements have been applied to estimate the ice content below freezing point [34,35,36,37,38]. To estimate the ice content of mortar sample saturated with NaCl solutions, the relationships between the combined effects of saturation degree, temperature and solution ions concentration and the electrical conductivity of mortar samples were clarified [37]. In addition, Wang et al. [39] attempted to use the maximum electrical resistivity (at lowest temperature) to evaluate the frost damage of cement paste immersed to water with FTCs. However, under the lowest temperature, the electrical resistivity changes not only with pore size but also with the saturation degree whose contributions are difficult to distinguish. On the one hand, frost damage increases the porosity [40] and connectivity, thereby decreasing the electrical resistivity. Frost damage causes water to move from the smaller pores to the larger pores and freeze in these large pores at the lowest temperature, thereby reducing the movable water content; the decrease in the saturation degree (amount of liquid water) increases the electrical resistivity. Therefore, in this stage, simply using the electrical resistivity at lowest temperature as the index cannot evaluate the frost damage degree accurately. By using the turning points from the logarithm plots of electrical resistivity versus temperature, Wang et al. also determined the freezing point and thawing point [41]. As found, the freezing point increases with the FTC, while the thawing point decreases. It seems that the freezing and thawing points change can also be used to evaluate the frost damage. However, a quantitative relationship has not been developed. Alternatively, Farnam et al. [42] studied the electrical response of mortar saturated with different concentrations of NaCl solution under FTC. The electrical resistivity change at the reference temperature (20 °C) was regarded as the damage index, but only the first FTC was considered. Because the authors used macro-scale samples for testing, the salt-frost damage may not be uniform along the depth of sample. The results from pulse velocity were used for comparison rather than that from mechanical tests. Besides, McCarter et al. [35] concerned the activation energy and depression in both freezing and thawing points for different cement-based materials under FTCs because these parameters are related to the microstructure of the porous media. Recently, Kim et al. [13] further studied these parameters by complex impedance with regard to a wide range of frequency and temperature. It is possible to evaluate the frost damage based on these parameters. However, their relationship with mechanical degradation was also not developed yet. Evaluation of the salt-frost damage of mortar via FTCs requires further investigation.

The main purpose of this paper was to examine various electrical characteristics and their correlation to the mechanical degradation of mortar saturated with NaCl solutions during FTCs, and then to propose a damage index for evaluating the salt-frost damage of specimens. Therefore, this study investigated the electrical, mechanical and microstructural responses of mortar saturated with deionized (DI) water and 5 and 15 wt.% NaCl solutions under FTCs. The thickness of specimen in the meso-scale was chosen because the pore solution concentration and the frost damage degree could be considered as uniform in the specimen with this scale. Based on the microstructural change results, the damage index from electrical measurements is proposed and discussed. To verify the reliability of the proposed damage index, the results from the three-point bending tests of normalized elastic modulus were compared with the damage index with FTCs. From the results of this study, the electrical measurement can be used for laboratory testing to estimate mechanical properties with a non-destructive way, which is useful for other researchers. Although the application of electrical measurements remains limited for evaluation of concrete structures in practice due to the influence from many factors, it has great potential and could be a useful tool.

## 2. Test Programs

Meso-scale mortar specimens (70 × 30 × 5 mm) were used in this experimental program and the details of test samples preparation procedures have been reported in our previous study [37]. As presented in Table 1, mix proportions with different water-to-cement ratios (W/Cs) were used based on ACI 211.1 [43], in which the coarse aggregates were excluded. Following the previous study [44], the large W/C difference was chosen to show difference in frost damage. Since water content plays an important role in frost damage of concrete, the water amount was kept constant in the mix proportions to avoid the confusion of damage contribution by water amount and W/C [45]. To promote the frost damage, the air-entraining agent was not included in the mixture. The ordinary Portland cement with a density of 3.16 g/cm^3^ was used. The density of fine aggregate is 2.67 g/cm^3^ and its maximum size is 1.2 mm. Firstly, the specimens were casted into 40 × 40 × 160 mm form and cured for 24 h before the form was removed. After demolding, the specimens were cured at a water tank for 90 days in a room temperature around 23 °C. Then the specimens were cut into a size of 70 × 30 × 5 mm by a wet saw. The meso-scale size was chosen because the pore solution concentration could be assumed to be uniform in the specimen and salt-frost damage could be quantitatively understood. The porosity and density of specimens are also shown in Table 1.

**Figure 1:**
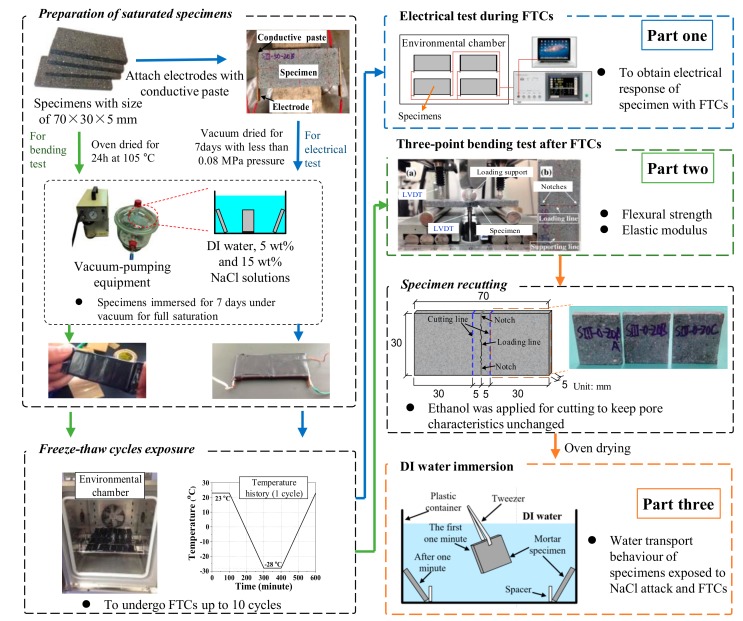
Schematic of the test procedures (for the colors of arrows, blue shows the procedure for electrical test; green shows the procedure for three-point bending test; and orange shows the procedure for deionized (DI) water immersion test) [11,37,47,48].

The test involves three parts, as shown in Figure 1: (1) Electrical test, (2) three-point bending test, and (3) water absorption test. The details of preparation procedures of electrical test samples can be found in Ref. [37]. After the mortar samples were prepared, the electrodes were attached on its two sides with conductive paste, as can be seen in first part of Figure 1. Then the specimens were cured for about one day. When the conductive paste was cured, the epoxy resin was also used to cover the electrode and conductive paste. In this case, the interface bonding between electrodes and specimens can be solidified. After curing of the epoxy resin for one week, the samples were dried in a vacuum desiccator for another week and kept drying until testing. The vacuum pressure was less than 0.08 MPa [46]. Because the adhesive properties of the conductive paste may be sensitive to elevated temperature, to avoid the detachment of electrode and mortar, instead of using oven drying, the vacuum drying method was used for the electrical tests. With the aid of vacuum desiccator, the samples were fully saturated with DI water, 5 and 15 wt.% NaCl solutions, respectively. Then they were sealed with saran and tape to prevent moisture transfer. The prepared specimens were placed into an environmental chamber to undergo FTCs; the freeze–thaw temperature history of the chamber is shown in Figure 1. The temperature was first kept at 23 °C for 100 min, then it decreased by 0.25 °C per min until the minimum temperature −28 °C and remained constant for 100 min, and then it rose by 0.25 °C per min until the maximum temperature of 23 °C. The freeze–thaw cycle was repeated 10 times. The above-mentioned temperature cycles are normally used for laboratory test. Since the purpose was not to predict the salt-frost damage in practical cases but to evaluate the mechanical properties after damage, 10 cycles were enough for this study. The electrical responses were recorded with the FTCs. In the electrical test, alternating current with two-point method was applied by a HIOKI LCR meter (IM3533-01). The voltage was 1V, which was chosen as an intermediate value based on existing studies [21,35]. The frequency of the alternating current can affect the resistivity results significantly [26]. As investigated by Merioua et al. [23], to assure the ohm’s law is valid, the range of appropriate frequencies is 500 Hz–50 kHz. Additionally, the polarization effect can greatly affect the resistivity and it is highly dependent on frequency; to reduce the effect to be minimum, the frequency used in this study was 1 kHz, which is the optimized frequency reported by McCarter et al. [35] on the study of concrete under FTCs. The test data was collected every 10 s by PC automatically. The electrical resistance of the conductive paste was around 0.5 Ω under FTCs, and it is ignorable compared with the electrical resistance of mortar sample. With regard to the geometrical effect, the electrical resistivity of the sample is

(1) $$\rho =\frac{AR}{L},$$

where *R* is the resistance of sample, *A* is the cross-sectional area and *L* is the length of the specimen.

The preparation of the three-point bending test sample is similar to that for the electrical test, excluding the parts for the electrode, the epoxy resin and specimens drying; the details can also be found in our previous report [11,48] and the procedures are also presented in the first part of Figure 1. After the three months of curing, the specimens were cut to the designed dimension. The cutting process was lasting for about one month. Three specimens for each case were prepared. Then the specimens were oven dried for 24 h at 105 °C. After that, the dried specimens were immersed for 7 days under vacuum for full saturation. In our previous study [47], without using vacuum pressure, the specimens of the same size with W/C = 0.3 took about 10 h to get fully saturated and the time was shorter for specimens with higher W/C. So we assume the specimens in this study are fully saturated. Next, the specimens were surface dried quickly by using a towel paper. To prevent moisture transfer, saran was used to covering the specimen and the vinyl/mastic tape was further applied for sealing. After the preparation of the fully-saturated samples, they were placed in the environmental chamber and undergo the same temperature history as the electrical test. After certain repeated FTCs, the specimens were removed from the environmental chamber and then unsealed. After labeling and making the notches on the two sides of specimens, they were kept at room temperature in a vacuumed chamber before performing the three-point bending test. To assure the direction of cracking, the notches on both sides of specimens were produced before the test, which is referred to the Standard (JCI-S-001-2003) [49]. This mechanical test method has been used by many researchers for the specimens at the meso-scale level and their test results show reasonable values [44,48,50], which verified the reliability of this method. Even though the mechanical properties of mortar in meso-scale could be different from in macro-scale, the purpose of the mechanical test is to understand the severity of salt-frost damage, which is the ratios of mechanical properties before and after FTCs, the relative values (ratios) will not be changed due to the size when the damage degree is uniform in the specimens. The mechanical test method is adopted in this study. After the three-point bending test, both the flexural strength and the elastic modulus were calculated based on JCI Standard [49] using the following equations:

(2) $$\sigma =\frac{3Pl}{2b_{0}h^{2}};\;E=\frac{P_{1/3}l^{3}}{4\delta_{1/3}bh^{3}}$$

where *σ* is the flexural strength in MPa, *E* is the elastic modulus in MPa, *P* is the maximum load in N, *l* is the bending span in mm, *b*_0_ and *b* are the width of the specimen at the loading line with and without a notch (mm), respectively, and *h* is the thickness of the specimen in mm. *P*_1/3_ is the one third of the maximum load, and *δ*_1/3_ is the deflection of the specimen at one third of the maximum load.

For the specimens saturated with 5 wt.% NaCl (W/C = 0.5), after the three-point bending test, the remaining part of the specimen was recut to the size of 30 × 30 × 5 mm specimens for the water absorption test. To cut the specimen with a diamond saw, the liquid is required to make the saw wet to reduce the damage of specimens. Since the specimens were saturated with NaCl solution and had salt-frost damage, if the water was used to cut the specimens, the remaining NaCl and CH in the pores may be resolved and change the microstructure. Ethanol was applied during cutting to keep the pore characteristics unchanged because the inorganic materials could not be resolved in the organic liquid. To avoid the further damage, the specimens were dried in an oven at 50 °C for 7 days. Next, the dried weights of specimens were measured using a scale at an accuracy of 0.1 mg. During the first minute, the specimen was grasped by tweezers. In this manner, the specimen could be fixed to a certain place and easily taken out from solution. As a result, the time that the specimen is immersed in water can be exactly controlled; the absorption test procedures followed our previous study [47]. Referred to the principle of ASTM standard C1585 [51], the interval time of measurement is increasing with absorption period (3, 6, 10, 15, 30, 60 s and 2, 3, 5, 10, 15, 20, 40, 60 min) since the solution absorption rate is decreasing. Because the size of our samples was too small, the absorption test did not simply follow the time in the ASTM standard C1585. Besides, instead of using surface absorption in the standard, the full immersion of the specimen was adopted in this study because the thickness of the specimen was quite small. Although the geometric effects may affect the amount of water that is absorbed, because all the specimens had the same dimension and the same testing method was applied, the pore size distribution change due to the salt-frost damage can still be understood by the test results. Based on the water absorption capacity, with regard to the size of samples, the water uptake amount q (g/g) may be determined by the dried mass of the sample:

(3) $$q=\frac{m-m_{d}}{m_{d}}$$

where *m* is the mass of specimen having absorbed solution, and *m_d_* is the mass of the dried specimen.

## 3. Test Results and Discussion 

**Figure 2:**
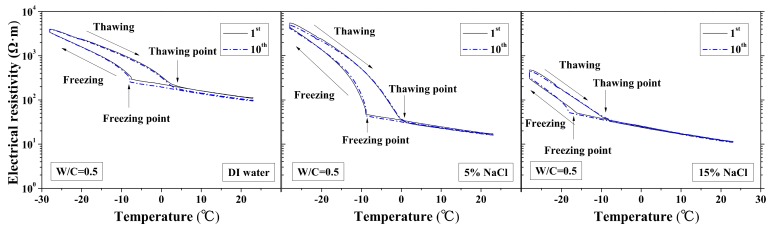
Electrical resistivity changes with temperature under first and tenth freeze–thaw cycles (water to cement ratio (W/C) = 0.5) [1].

The changes in the electrical resistivity of specimens with temperature during FTCs were previously reported in [37], as shown in Figure 2. The test results were further analyzed for comparison of each of the electrical characteristics, including electrical resistivity at reference temperature and the lowest temperature, activation energy, and freezing and thawing points.

### 3.1. Electrical Resistivity Values at Reference Temperature and Lowest Temperature

**Figure 3:**
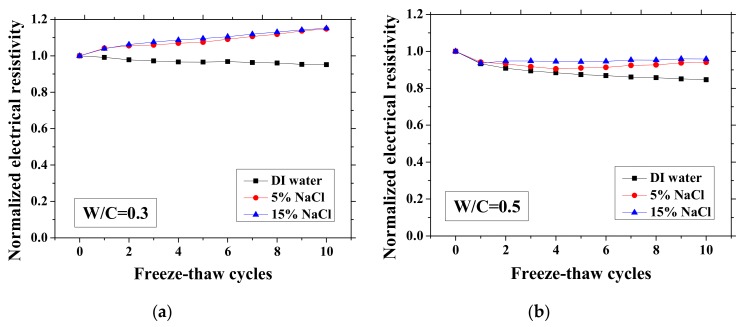

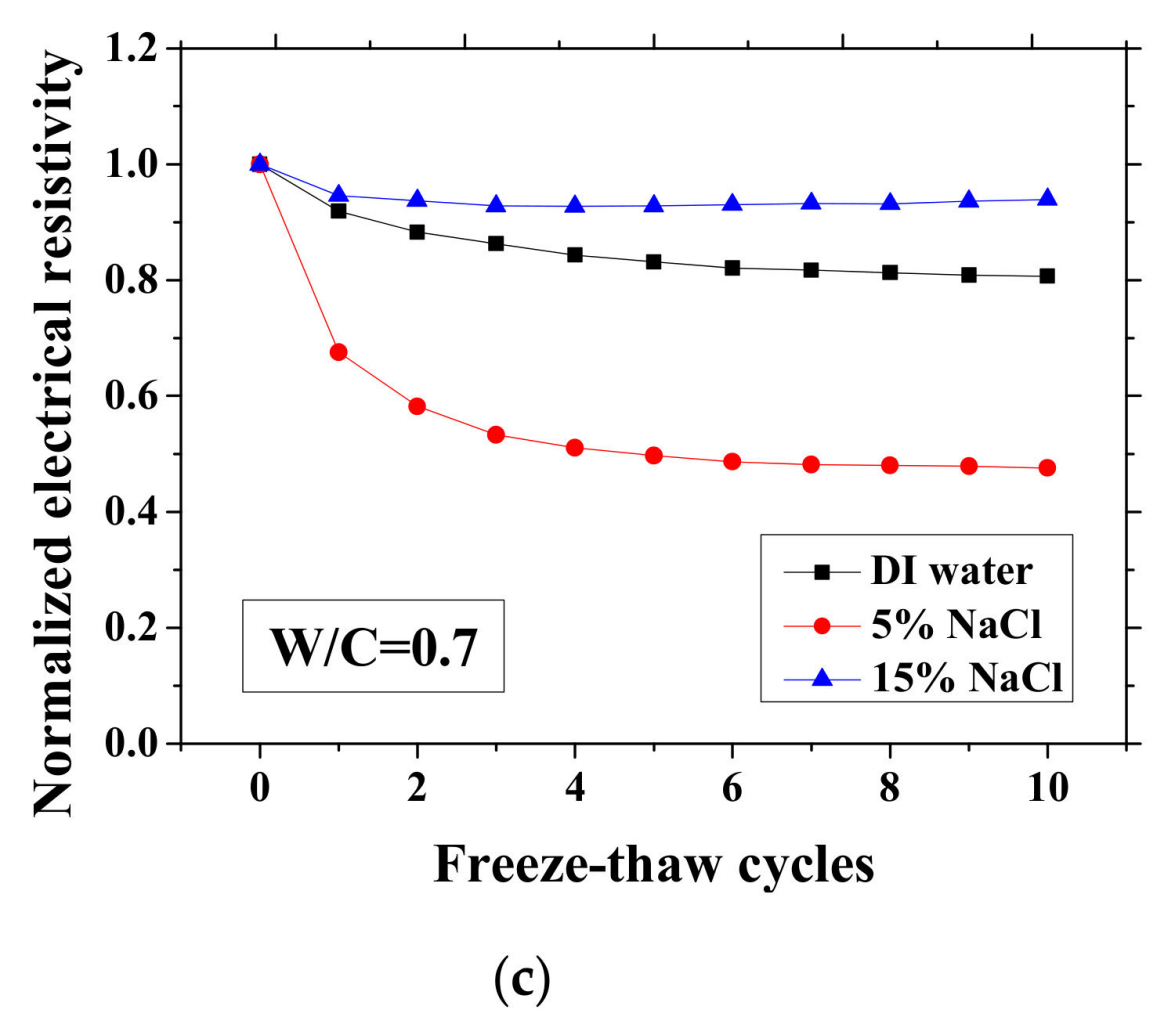
Changes in the electrical resistivity at the reference temperature (23 °C) with freeze-thaw cycles (FTCs): (**a**) W/C = 0.3, (**b**) W/C = 0.5 and (**c**) W/C = 0.7.

**Figure 4:**
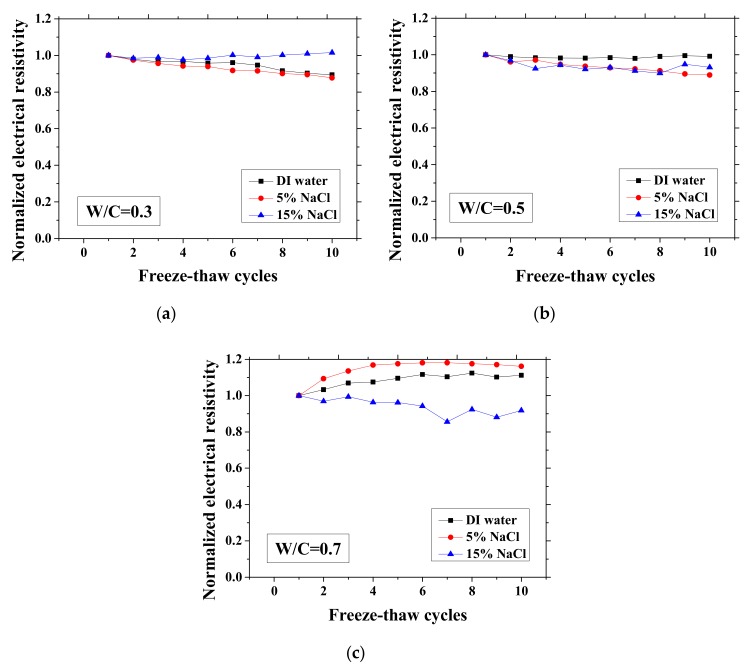
Changes in the electrical resistivity at the lowest temperature (−28 °C) with FTCs: (**a**) W/C = 0.3, (**b**) W/C = 0.5 and (**c**) W/C = 0.7.

According to the study of Farnam et al. [42], the change of the electrical resistivity at the reference temperature before and after FTCs can be adopted to evaluate the salt-frost damage of mortar since the resistivity will decrease after FTCs due to the damage, whereas Wang et al. [39] regarded the maximum electrical resistivity as the parameter of frost damage because it can linearly increase with FTCs. Therefore, in this paper, as shown in Figure 3 and Figure 4, the normalized electrical resistivity at temperatures 23 and −28 °C were considered, respectively. The normalized electrical resistivity is given by the ratio of the electrical resistivity with FTCs to the electrical resistivity at the first cycle. The electrical characteristics were examined to correlate with the mechanical property reduction under FTCs. The values of the electrical resistivity at the reference temperature (23 °C) and the lowest temperature (−28 °C) at first cycle are presented in Table 2. The discussion of the results in Table 2 can be seen in [37].

As presented in Figure 3 and Figure 4, the electrical resistivity of mortar at 23 and −28 °C generally decreases with frost damage because the frost action can cause increases in porosity and connectivity. However, it also shows different tendency compared with previous studies [39,42] due to the existence of NaCl. For 5 and 15 wt.% NaCl solution-exposed samples with W/C = 0.3, contrasting tendencies were observed at 23 °C (Figure 3a), indicating that the frost damages in these cases were insignificant because they have lower amounts of moisture and salt and higher initial strength before FTCs, than the cases of W/C = 0.5 and 0.7, due to the larger cement amount. Moreover, for the samples (W/C = 0.3) saturated with NaCl solutions, the chloride binding and further hydration could reduce the content of movable ions and result in the increase in the electrical resistivity. Even though the chloride binding occurs and affects the electrical resistivity of samples of W/C = 0.5 and 0.7 (see Figure 3b, c), the salt-frost damage was so severe that the electrical resistivity shows a decreasing tendency. An increasing tendency was also observed at −28 °C in the cases of DI water and 5 wt.% NaCl solution-exposed samples with W/C = 0.7 (see Figure 4c). Unlike the cases at 23 °C, the increase in electrical resistivity at −28 °C was caused by more severe frost damage. Since the frost damage in the case of 15 wt.% NaCl was not so severe, it does not show an increasing tendency. The pore connectivity and porosity change can influence on the electrical resistivity at lowest temperature. Owing to the higher connectivity, the water can flow more freely at low temperature. On one hand, the movable water can contribute to the flow of ions, which decrease the electrical resistivity. Since the samples with W/C = 0.7 had high connectivity before the FTCs due to the more porous nature, the contribution from the connectivity change may be not obvious, whereas it could be the dominant effect for the electrical resistivity decrease in the cases of W/C = 0.3 and 0.5. On the other hand, the increase of connectivity could promote the water from the small pores moving to the larger pores. It can facilitate the ice nucleation and increase the amount of ice content. Besides, because of the severe damage, the increase in the porosity can provide a larger space for ice crystallization and yield the reduction in the saturation degree, thereby increasing the electrical resistivity at −28 °C.

**Figure 5:**
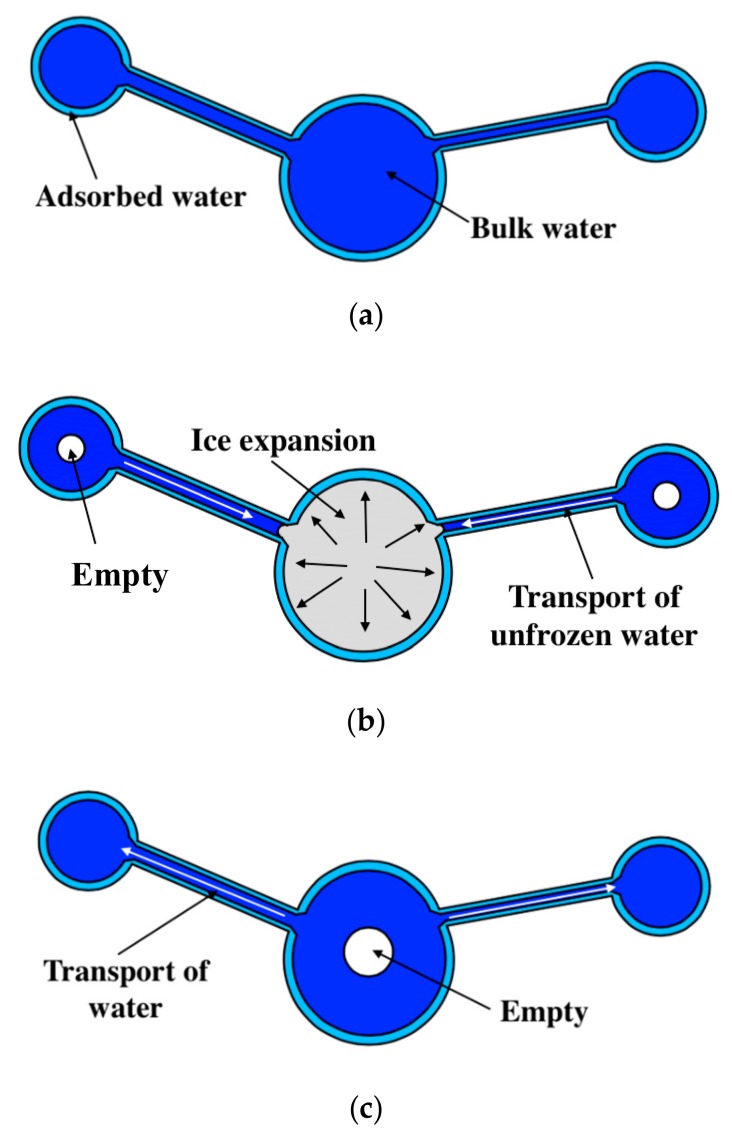
Water transport mechanism during FTC: (**a**) Before FTC, (**b**) during freezing and (**c**) after FTC.

Based on the studies in [5,33,41,52], the water transport mechanism during FTC is clarified for fully-saturated specimens (see Figure 5). During freezing, the ice expansion can cause residual deformation, resulting in the increase in the pore volume. Subsequently, more unfrozen water from smaller pores will move to the ice front and contribute to ice formation [41]. With the FTCs, the damage would develop, resulting in an increasing amount of ice in the larger pores and a decreasing amount of bulk water in the smaller pores. Moreover, the superficial area of pores would increase with the frost damage development, and the amount of adsorbed water could increase as well because it is highly dependent on the superficial area [52]. Because the total water content is constant, the movable water at the lowest temperature can be reduced. In this regard, the electrical resistivity at the lowest temperature may increase. However, because of the hydraulic pressure, both the porosity and the connectivity of pores increase. Thus, the electrical resistivity can also decrease (see Figure 4).

### 3.2. Activation Energy Changes with FTCs

**Figure 6:**
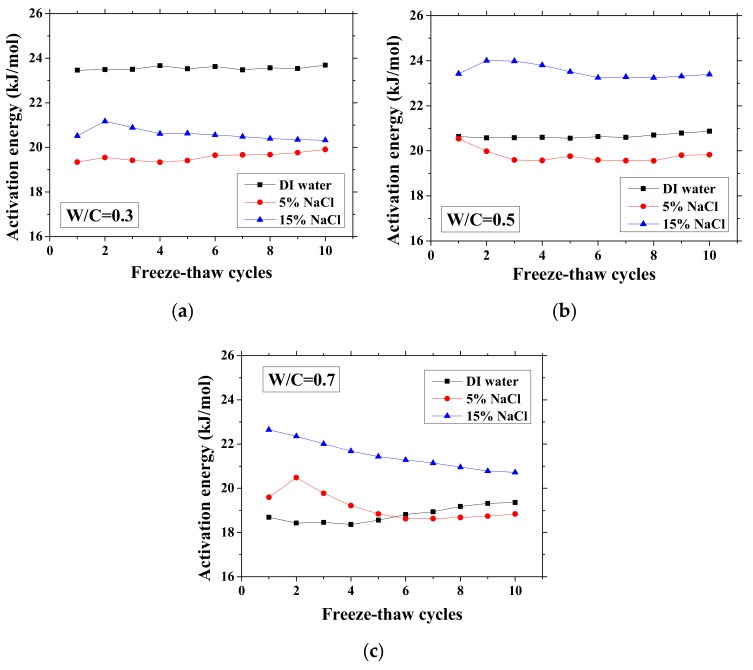
Changes in the activation energy of specimens with FTCs: (**a**) W/C = 0.3, (**b**) W/C = 0.5 and (**c**) W/C = 0.7.

The activation energy for conduction is a parameter that can quantify the sensitivity of electrical resistivity of a mortar sample to temperature. This activation energy is correlated to not only the pore characteristics and pore solution compositions [27] but also the saturation degree of samples [25]. Since the activation energy is mainly correlated to the microstructure properties [27,35], it has potential to be used for evaluation of salt-frost damage. When the microstructure properties changed, the mechanical properties change as well [40]. For partially-saturated samples, the activation energy can be calculated based on the activation energy of fully-saturated samples and the saturation degree [36]. If the frost damage degree is known, then the saturation degree change due to the porosity increase as well as its effect on activation energy could be understood. Because the microstructure properties change can affect the activation energy, with FTCs, the frost damage induced porosity change may be analyzed by the activation energy. However, for the cases with the NaCl solution, the chemical reaction could occur with the FTCs. Thus, because the activation energy change is rather complicated, the test results are important for further understanding of the relationship between the activation energy and the salt-frost damage degree. Based on the electrical test results, the activation energy of samples change with FTCs can be obtained, as shown in Figure 6. The activation energy was calculated as below [25,36]:

(4) $$E_{a}=-\frac{R_{g}\ln\left(\frac{\sigma_{T}}{\sigma_{ref}}\right)}{\frac{1}{T}-\frac{1}{T_{ref}}},$$

where *E_a_* is the activation energy for conduction (kJ/mol), *R_g_* is the universal gas constant (8.314 J/mol/K), *σ_T_* is the electrical conductivity of mortar sample at the absolute temperature *T* (K), *σ_ref_* is the electrical conductivity of mortar sample at the absolute reference temperature *T_ref_* (K). In this study, the reference temperature is 23 °C (296.14 K).

Without exposure to FTCs, for DI water cases, the activation energy decreases with W/C because it decreases with moisture content [25]. The higher the W/C is, the higher the volume ratio of moisture that samples can contain is. With regard to the cases of the NaCl solution concentration difference, the activation energy change was inconsistent for different W/Cs. In the cases of W/C = 0.7, the activation energy increases with NaCl solution concentration, possibly because of the formation of Friedel’s salt, which can consume movable water and ions. For samples exposed to higher concentration NaCl solution, the absorbed moisture content is lower because the newly-formed Friedel’s salt can fill the pores [45] and because the amount of Friedel’s salt increases with NaCl concentration [47]. In contrast, for the other cases, although the concentration of remaining pore solution is high, the activation energy is low. It is very likely that the amount of Friedel’s salt crystallization in the cases of W/C = 0.3 and 0.5 is low [48]. The activation energies of the two cases saturated with 5 wt.% NaCl are similar, whereas for the 15 wt.% NaCl cases, the difference becomes clear because of the larger amount of salt crystallization and reduced amount of moisture content. 

As observed, there is no general tendency for the activation energy changes with FTCs. The change tendency varies with the W/C and concentrations of NaCl solution. According to the results, the variation of activation energy caused by FTCs is not significant in the case of W/C = 0.3 and 0.5; however, the decrease in activation energy in the case of W/C = 0.7 and 15 wt.% NaCl solution-saturated sample cannot be simply neglected. After frost damage, the degree of saturation decreased because the deformation and cracks induced the connection of air voids and increased the volume of air voids, resulting in higher activation energy. Moreover, it is known that the activation energy of mortar increases with the reduction in water saturation degree [25]. The actual saturation degree decreases with frost damage, as described in Section 3.1. Nevertheless, the increase in porosity caused by frost damage can result in a decrease in the activation energy.

**Figure 7:**
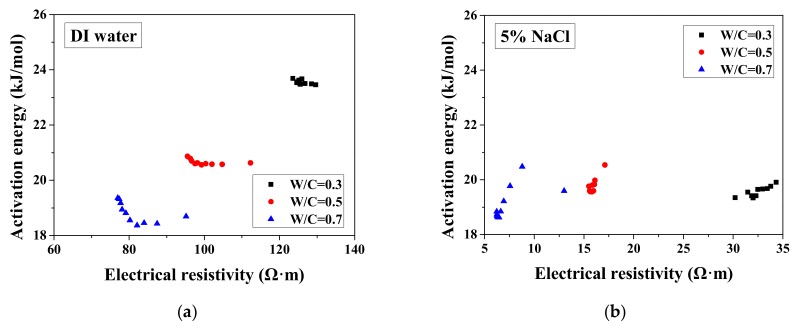

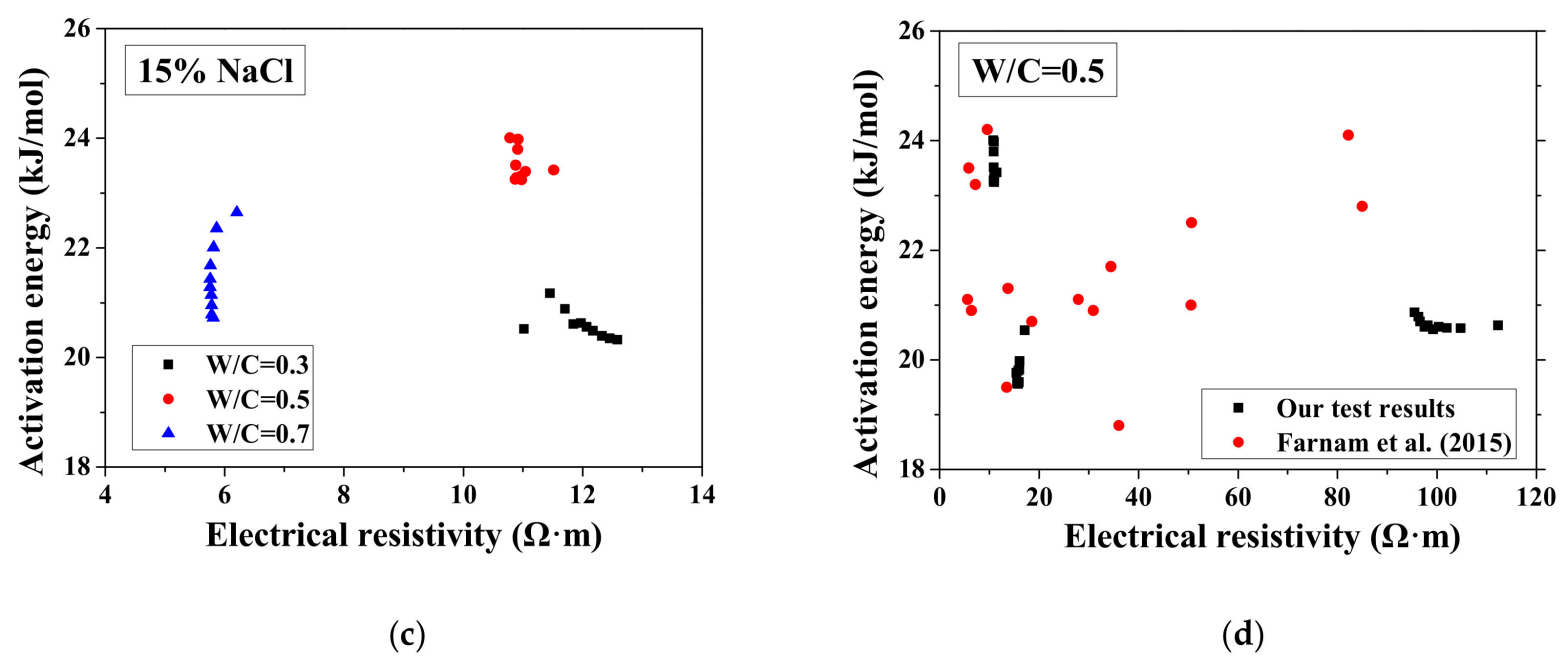
Changes in the activation energy of specimens with electrical resistivity: (**a**) DI water, (**b**) 5 wt.% NaCl, (**c**) 15 wt.% NaCl and (**d**) W/C = 0.5.

According to the study of Liu and Presuel-Moreno [27], for DI water saturated concrete samples, there is a relationship between the activation energy and the electrical resistivity at the reference temperature. In this case, once the electrical resistivity at the reference temperature decreases because of the frost damage, the activation energy would decrease correspondingly. The trend was observed from the study of McCarter et al. [35]. However, the relationship between activation energy and electrical conductivity cannot be observed from our test results and the test results from Farnam et al. [42], especially for higher NaCl concentration solution (see Figure 7), possibly because the specimen saturation degree changed during FTCs with frost damage development. How the activation energy at full saturation changes with FTCs remains unclear and requires further investigation.

### 3.3. Change in Freezing and Thawing Points with FTCs

**Figure 8:**
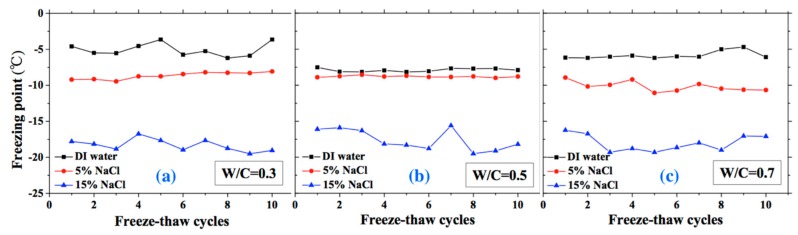
Changes of the freezing point with FTCs: (**a**) W/C = 0.3, (**b**) W/C = 0.5 and (**c**) W/C = 0.7.

**Figure 9:**
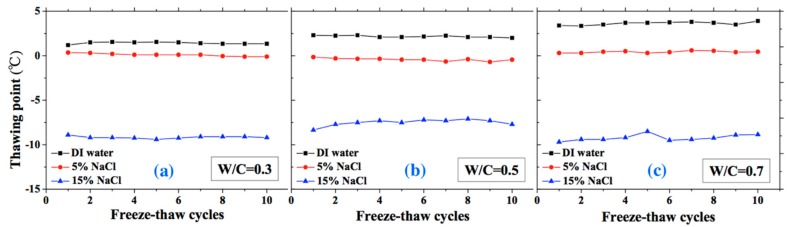
Changes in the thawing point with FTCs: (**a**) W/C = 0.3, (**b**) W/C = 0.5 and (**c**) W/C = 0.7.

The freezing and thawing points with FTCs were analyzed from the test results, as shown in Figure 8 and Figure 9, respectively. The electrical resistivity was changing with temperatures, when the ice was crystallized or melted at certain temperature, the amount of movable ions could be changed significantly, as well as the electrical resistivity. The temperatures corresponding to the sudden change of electrical resistivity could be regarded as freezing or thawing points, as shown in Figure 2 [37]. The freezing point of solution in mortar depends on the pore size and solution concentration. After frost damage, the porosity and connectivity increase (i.e., the pore size can increase). Consequently, the freezing and thawing points may increase, as shown in [39]. However, as Figure 8 shows, no obvious freezing point change tendency can be observed. The variation in freezing point with FTCs may be caused by the heterogeneous ice nucleation nature [53]. The freezing and thawing points decrease with the NaCl solution concentration. With different W/C, the trend is not clear. The case of W/C = 0.5 has the lowest freezing point in terms of the DI water case, whereas the freezing points of 5 and 15 wt.% NaCl cases are similar for different W/C.

The thawing point of sample was found to be steady compared with freezing point. For the DI water case, the thawing point increases with W/C (i.e., the macro-pore size is larger in higher W/C). However, for the 5 and 15 wt.% NaCl solution cases, this conclusion may not be appropriate because of the pore solution concentration and possible salt crystallization. When the NaCl penetrates into mortar, the formation of Friedel’s salt can occur and fill the pores, altering the pore size of mortar [47]. With increasing NaCl solution concentration and W/C, the amount of Friedel’s salt increases [48]. Therefore, for the 5 and 15 wt.% NaCl solution-saturated samples, the macro-pore size is not larger with higher W/C.

### 3.4. Mechanical Properties Change with FTCs

**Figure 10:**
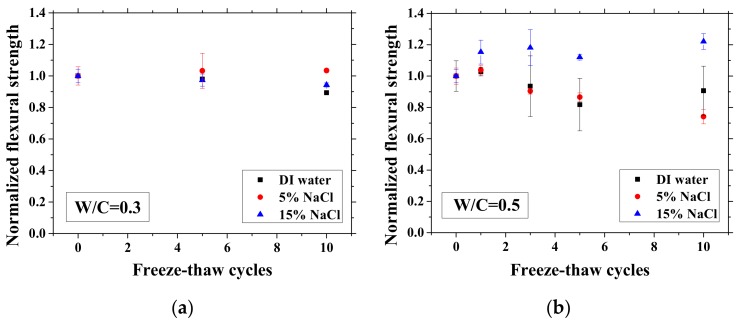

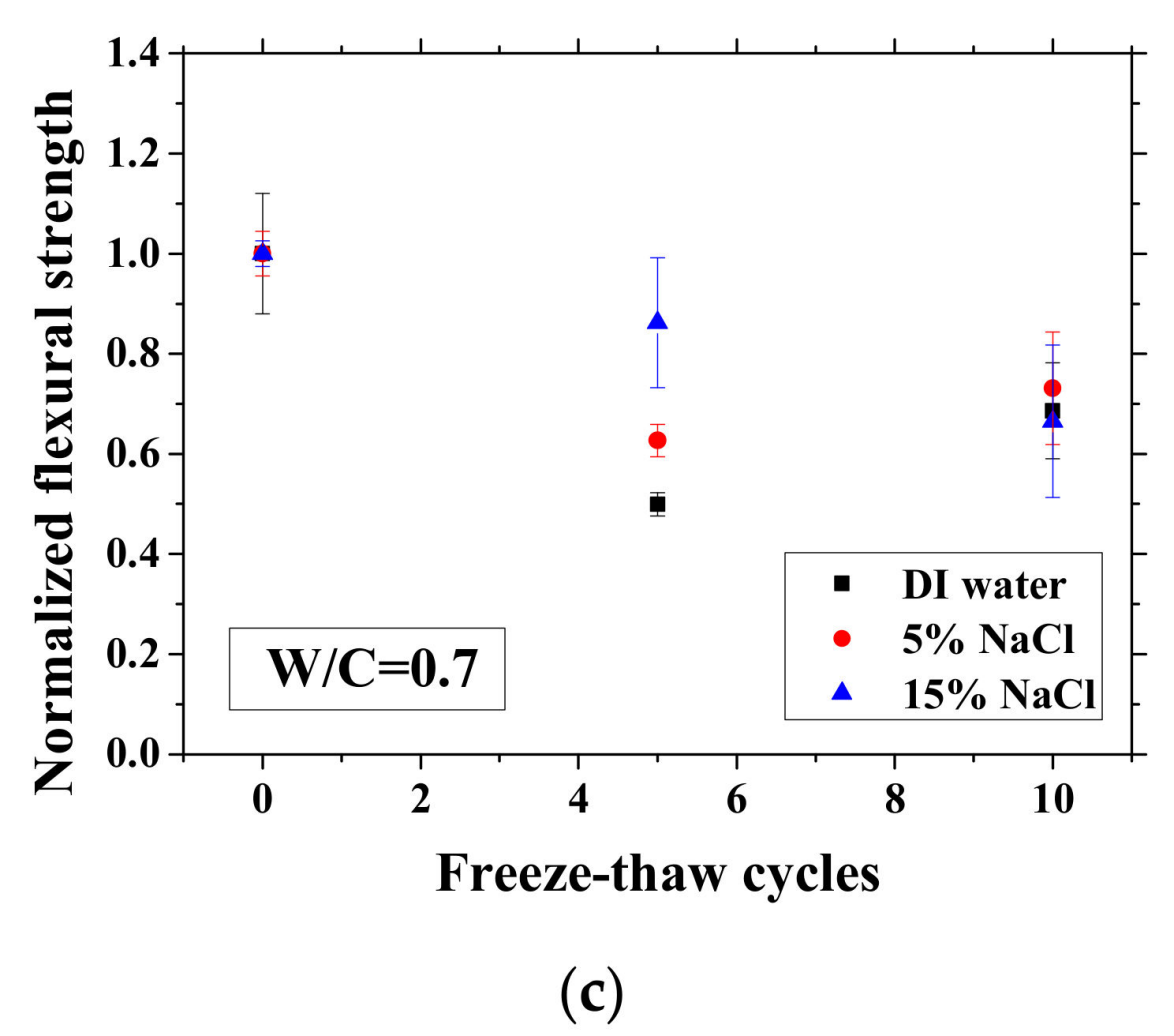
Changes in the normalized flexural strength with FTCs: (**a**) W/C = 0.3, (**b**) W/C = 0.5 and (**c**) W/C = 0.7.

**Figure 11:**
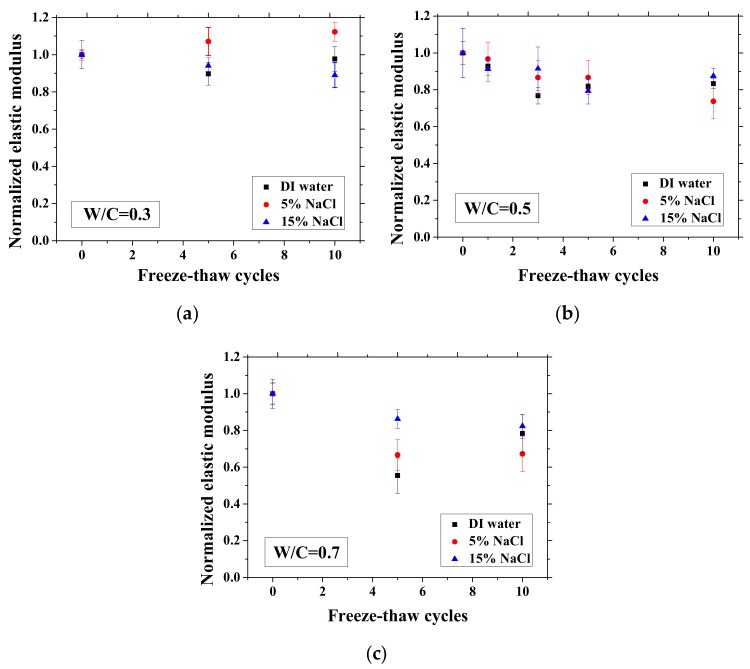
Changes in the normalized elastic modulus with FTCs: (**a**) W/C = 0.3, (**b**) W/C = 0.5 and (**c**) W/C = 0.7.

After certain FTCs, the samples were removed from the environment chamber, and then the three-point bending test was conducted. The flexural strength and elastic modulus were calculated based on Equation (2). The ratio of flexural strength and elastic modulus of the sample after FTCs to that before FTCs is defined as the normalized flexural strength and elastic modulus, respectively. The results are shown in Figure 10 and Figure 11. In each case, the average value with an error bar from three samples is presented. From the figures, the mechanical degradation can be observed clearly for W/C = 0.5 and 0.7, which is important to understand the severity of salt-frost damage. In particular, the 5 wt.% NaCl case reduced progressively with FTCs finally has the most severe damage. This process can be explained by the combined effect of ice nucleation and salt crystallization [7]. The ice expansion can cause destructive damage in mortar and reduce its flexural strength and elastic modulus. However, the amount of ice in the case of 5 wt.% NaCl is less than the case of DI water in theory [37] and thus cannot cause such severe damage. According to the study of Wu et al. [7], after salt-frost damage of concrete occurs, the Friedel’s salt was detected. Thus, it was believed that this salt crystallization also contributes to the damage of mortar in addition to the ice formation.

### 3.5. Relationships between Normalized Elastic Modulus and Normalized Electrical Resistivity (23 °C) and between Normalized Electrical Resistivity (−28 °C) and Activation Energy

**Figure 12:**
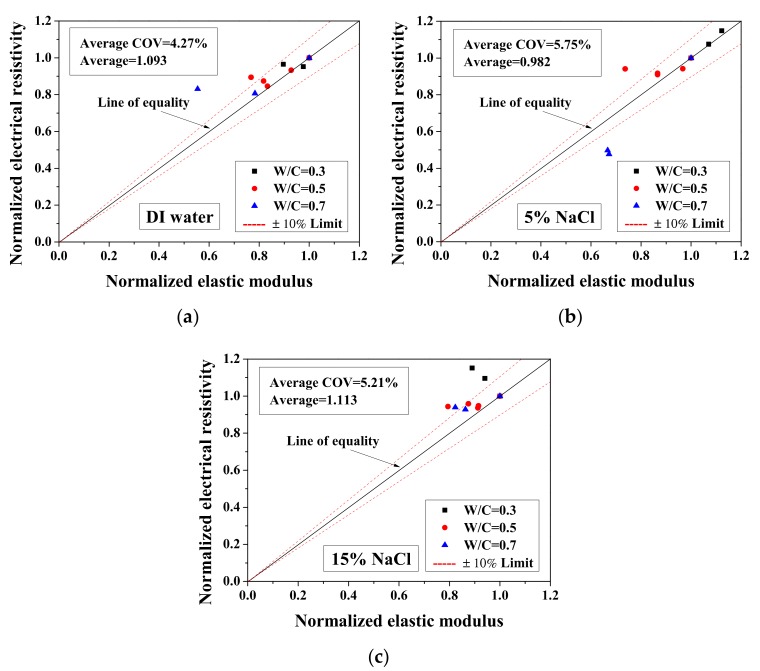
Comparison of the normalized electrical resistivity at the reference temperature (23 °C) and the normalized elastic modulus for different cases: (**a**) DI water, (**b**) 5 wt.% NaCl and (**c**) 15 wt.% NaCl.

**Figure 13:**
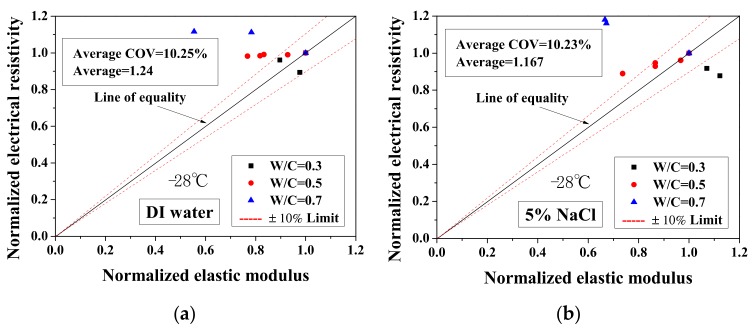

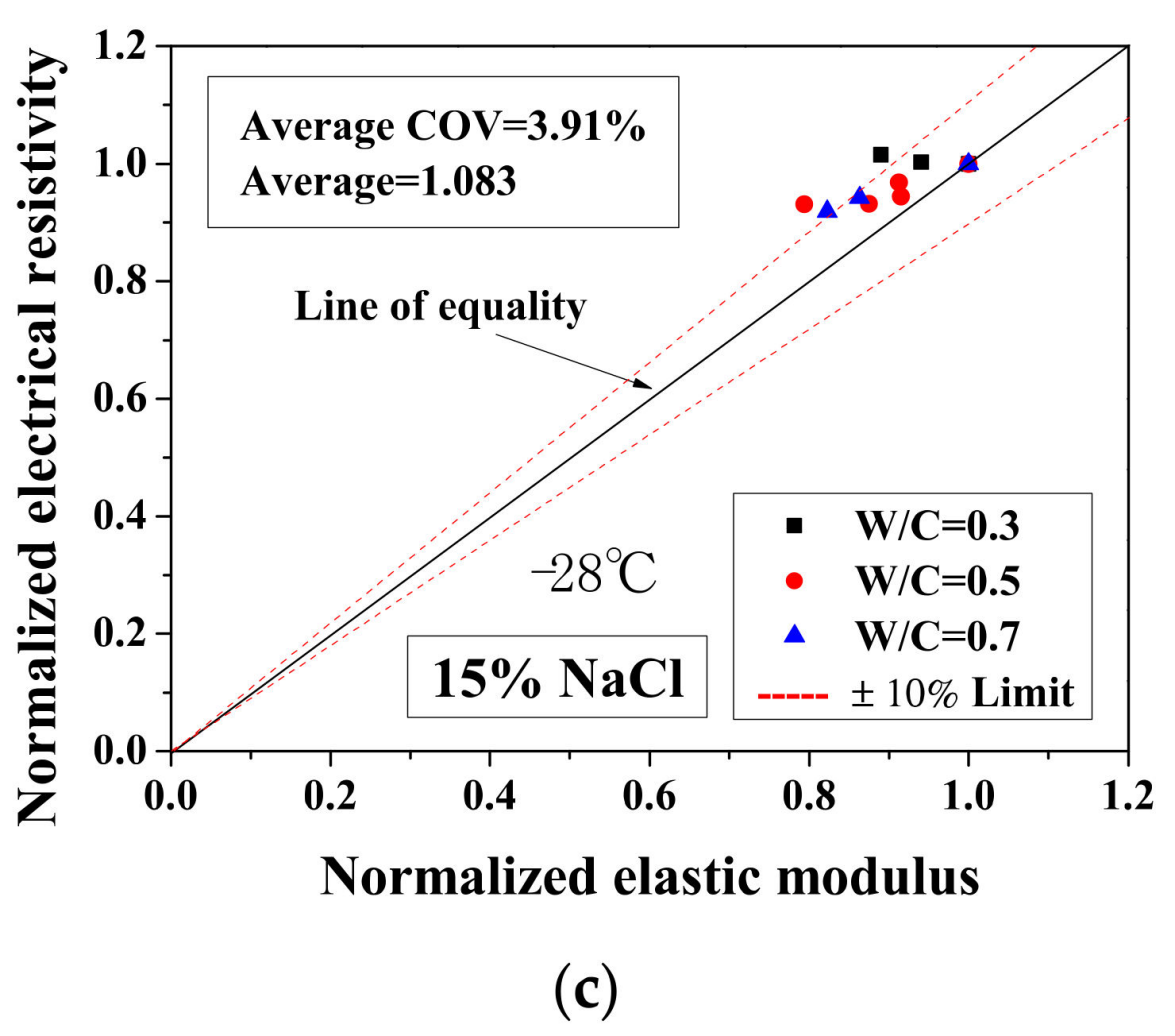
Comparison of the normalized electrical resistivity at the lowest temperature (−28 °C) and the normalized elastic modulus for different cases: (**a**) DI water, (**b**) 5 wt.% NaCl and (**c**) 15 wt.% NaCl.

**Figure 14:**
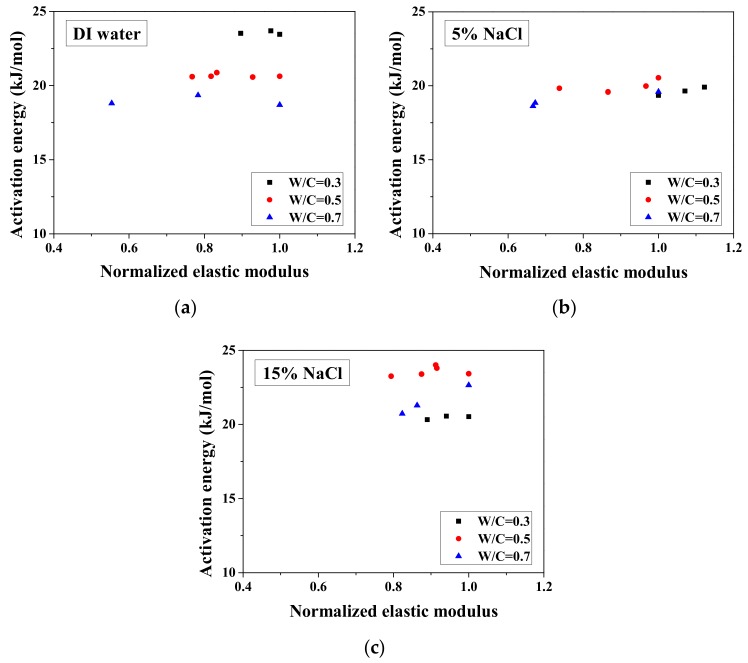
Comparison of the activation energy and the normalized elastic modulus for different cases: (**a**) DI water, (**b**) 5 wt.% NaCl and (**c**) 15 wt.% NaCl.

Because the electrical property changes are related to the mechanical degradation, the relationships between the normalized elastic modulus and the normalized electrical resistivity (23 °C) and between the normalized electrical resistivity (−28 °C) and the activation energy were studied. The comparisons of normalized electrical resistivity (23 °C) and normalized elastic modulus are shown in Figure 12. For the cases of DI water and 5 wt.% NaCl, these quantities were in good agreement. For the cases of 15 wt.% NaCl, although the variation is slightly larger, the evaluation method is acceptable for salt-frost damage. As shown in Figure 13 and Figure 14, while the normalized elastic modulus cannot agree with the normalized electrical resistivity (−28 °C), no clear relationship can be observed between the normalized elastic modulus and the activation energy. The detailed reason for the disagreement was discussed in Section 3.1 and Section 3.2 From Figure 12 and Figure 13, the accuracy of the evaluation method based on the normalized electrical resistivity can be seen.

### 3.6. Water Absorption Test Results

**Figure 15:**
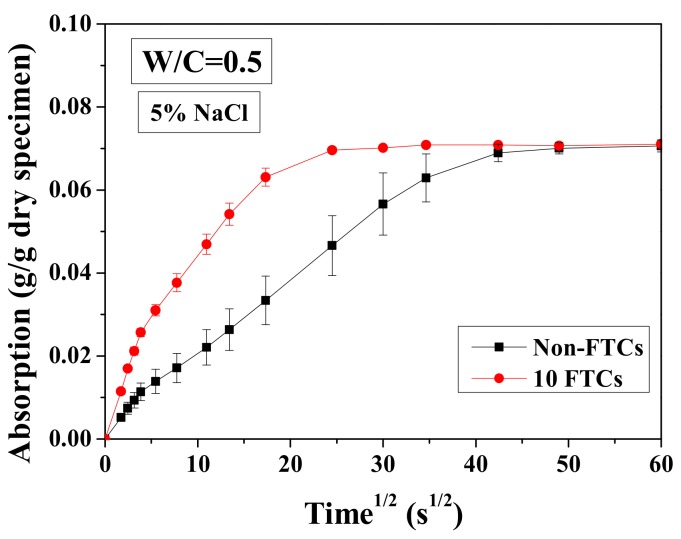
Water absorption test results of specimens saturated with 5 wt.% NaCl before and after 10 FTCs.

For a short period of immersion (1 h), the test results of cumulative solution absorption characteristics are presented versus the square root of time in Figure 15. The absorption amount was analyzed based on Equation (3), normalized by the dried mass of sample. In Figure 15, the water transport in specimens before and after 10 FTCs is shown. It is clear that the absorption amount of specimens varies insignificantly with FTCs. In contrast, the absorption rate is very different (i.e., the connection of specimens altered dramatically after FTCs). The absorption process may be divided into two stages: initial absorption and long-time absorption. During the initial absorption, the mass of the sample increased linearly and rapidly. This result may be attributed to the capillary pore absorption. If the pores can be assumed to be continuous tubes for solution transport, then the larger diameter can transport the solution more rapidly [54]. For the long-time absorption, the absorption curve is also linear. The gel pore solution absorption and filling of air voids may responsible for mass gain during this period. Because of the small size of gel pores, the solution transport inside could be rather slow, requiring a long time to be totally filled by water. The slope of the curve increases with FTCs. While the specimen without FTCs becomes apparently fully-saturated after around 40 min of immersion, for the specimens suffered 10 FTCs, they only need about 20 min. Therefore, after FTCs, there was an insignificant change for the porosity, whereas the connectivity clearly increased.

## 4. Comparison of the Damage Index and Change in the Mechanical Properties with FTCs

During freezing, because of the ice formation, micro-cracks or residual deformation could be induced. As a result, the specific surface of pores increases, and the actual saturation degree decreases. After the FTCs, the porosity and connectivity increase because of the frost damage, which can decrease the formation parameter of concrete. As defined by the other researchers [24,30,55], the formation parameter is the reciprocal of the porosity and the connectivity of the sample. Thus, the electrical resistivity change with FTCs is dependent on the damage degree of the specimens. Based on the electrical test results, the model for the damage degree was proposed.

For the fully-saturated samples, before exposure to the FTCs, the electrical conductivity of sample is determined by the electrical conductivity of the pore solution and the formation parameter [22,23] as below:

(5) $$\sigma_{ref,s,0}=\sigma_{ref,pore,0}/F_{0}=\sigma_{ref,pore,0}\cdot \phi_{0}\cdot \beta_{0}=\sigma_{ref,pore,0}\cdot \phi_{0}{}^{m},$$

where σ*_ref,s,_*_0_ is the electrical conductivity of the saturated sample before FTCs, σ*_ref,pore_*_,0_ is the electrical conductivity of the pore solution before FTCs, *F*_0_ is the formation parameter before FTCs, *ф*_0_ and β_0_ are the original porosity and the connectivity of the sample, respectively, and *m* is the fitting parameter. The formation parameter is the inverse of the porosity and the connectivity of the sample, and there is an intrinsic relationship between porosity and connectivity. According to Archie’s law [24], the formation parameter can be replaced by the porosity with a power law. From Merioua et al. [23], for mortar with W/C equal to 0.5 m is approximately 3.

After frost damage, the porosity and connectivity of specimen increase as the degree of saturation decreases. As a result, the electrical conductivity of specimens may not increase in a simple manner. Therefore, to quantitatively evaluate the frost damage, the electrical resistivity change caused by the saturation degree should be considered properly. Because of the increased porosity, the saturation degree of samples (*S*) decreases to:

(6) $$S=\frac{\phi_{0}}{\phi_{a}},$$

where *ф_a_* is the porosity after frost damage.

The water saturation degree can affect the electrical resistivity significantly [56], and the relationship could be written as:

(7) $$\frac{\sigma_{ref,p,0}}{\sigma_{ref,s,0}}=S^{n_{0}},$$

(8) $$\frac{\sigma_{ref,p,0}}{\sigma_{ref,pore,0}}=\frac{1}{F_{0}}S^{n_{0}}=\phi_{0}{}^{m}\cdot S^{n_{0}},$$

where *σ_ref,p,_*_0_ is the electrical conductivity of partially-saturated sample before FTCs; *n*_0_ is the saturation coefficient before FTCs (for mortar sample with W/C = 0.5, and n_0_ is approximately equal to 3.5 [22]), which is determined by the porosity, connectivity and chemical composition of the pore solution of the sample.

After the exposure to FTCs, by combining Equations (5)–(7), the measured electrical conductivity of sample is:

(9) $$\sigma_{ref,a}=\sigma_{ref,p,a}=\sigma_{ref,s,a}\cdot S^{n_{a}}=\sigma_{ref,pore,a}\cdot \phi_{a}{}^{m}\cdot S^{n_{a}}=\sigma_{ref,pore,a}\cdot \phi_{a}{}^{m}\cdot {\left(\frac{\phi_{0}}{\phi_{a}}\right)}^{n_{a}},$$

where *σ_ref,a_* is the electrical conductivity of the specimen at the reference temperature after FTCs, *σ_ref,p,a_* is the electrical conductivity of the partially-saturated specimen at the reference temperature after FTCs, *σ_ref,s,a_* is the electrical conductivity of the saturated specimen at the reference temperature after FTCs, *σ_ref,pore,a_* is the electrical conductivity of the pore solution at the reference temperature after FTCs and n_a_ is the saturation coefficient after the FTCs.

For NaCl solution cases, because of the change of the chemical phases (e.g., formation of Friedel’s salt) in the sample during FTCs, the electrical conductivity of the pore solution could be changed. According to Equations (5) and (9), the measured electrical resistivity of the sample is:

(10) $$\frac{\rho_{ref,a}}{\rho_{ref,0}}=\frac{\sigma_{ref,0}}{\sigma_{ref,a}}=\frac{\sigma_{ref,pore,o}\cdot \phi_{0}{}^{m}}{\sigma_{ref,pore,a}\cdot \phi_{a}{}^{m}\cdot {\left(\frac{\phi_{0}}{\phi_{a}}\right)}^{n_{a}}}=\left(\frac{\sigma_{ref,pore,0}}{\sigma_{ref,pore,a}}\right)\cdot {\left(\frac{\phi_{0}}{\phi_{a}}\right)}^{m-n_{a}},$$

where *ρ_ref,_*_0_ and *ρ_ref,a_* are the electrical resistivity of specimens at the reference temperature before and after the FTCs, respectively; and σ_ref,0_ is the electrical conductivity of the specimen at the reference temperature before FTCs.

In particular, for the DI water case, there is no chemical phase change in the sample during the FTCs, and the electrical conductivity of the pore solution is the same as that before FTCs. Thus, the normalized electrical resistivity at the reference temperature is:

(11) $$\frac{\rho_{ref,a}}{\rho_{ref,0}}=\frac{\sigma_{ref,0}}{\sigma_{ref,a}}=\frac{\sigma_{ref,pore,0}\cdot \phi_{0}{}^{m}}{\sigma_{ref,pore,0}\cdot \phi_{a}{}^{m}\cdot {\left(\frac{\phi_{0}}{\phi_{a}}\right)}^{n_{a}}}={\left(\frac{\phi_{0}}{\phi_{a}}\right)}^{m-n_{a}},$$

Obviously, the resistivity changes with both (*ф*_0_/*ф*_a_)^m^ and (*ф*_0_/*ф*_a_)^na^. By reversing Equation (10), the ratio of formation parameter can be given by:

(12) $$\frac{F_{a}}{F_{0}}=\frac{\phi_{0}{}^{m}}{\phi_{a}{}^{m}}=\frac{\sigma_{ref,pore,a}}{\sigma_{ref,pore,0}}\cdot \frac{\rho_{ref,a}}{\rho_{ref,0}}\cdot {\left(\frac{\phi_{0}}{\phi_{a}}\right)}^{n_{a}},$$

where *F*_0_ and *F*_a_ are the formation parameters of the specimen at the reference temperature before and after FTCs, respectively. 

Because the water content was not changed during the FTCs, in the study of Farnam et al. [42], the saturation was considered as a constant. Assuming the electrical resistivity of pore solution was not changed during the FTCs, the damage index could be considered as the change of electrical resistivity at reference temperature

(13) $$\mathrm{Damage}\;\mathrm{index}=1-\frac{F_{a}}{F_{0}}=1-\frac{\rho_{a}}{\rho_{0}},$$

where *F*_a_ (*ρ_a_*) and *F*_0_ (*ρ_0_*) are the formation parameter (electrical resistivity) at the reference temperature after and before FTCs, respectively.

For evaluation of mechanical property degradation, the change of porosity is needed. Since the formation parameter is an indicator of porosity and connectivity, it is reasonable to interpret the change in the formation parameter as the damage index. However, in the case of salt-frost damage, the relationship between the formation parameter and the electrical resistivity at the reference temperature is not linear, Equation (13) for damage index needs further improvement.

After salt crystallization, the movable ions in the pore solution could be reduced; thus, *σ_ref,pore,_*_0_ >*σ_ref,pore,_*_a_. Because of salt-frost damage, for specimens with W/C = 0.5 and 0.7, *ф*_0_/*ф*_a_ < 1. However, for the specimen with W/C = 0.3, because of its high strength and stiffness, there is almost no damage. Moreover, because crystallized salt fills part of the pores, *ф*_0_/*ф*_a_ can be higher than 1. For specimens saturated with NaCl solutions with W/C = 0.3, the normalized electrical resistivity can increase with FTCs, whereas the others decrease. In this case, considering Equation (12), the damage index could be given by:

(14) $$\mathrm{Damage}\;\mathrm{index}=1-\frac{F_{a}}{F_{0}}=1-\frac{\phi_{0}{}^{m}}{\phi_{a}{}^{m}}=1-\left(\frac{\sigma_{ref,pore,a}}{\sigma_{ref,pore,0}}\right)\cdot \left(\frac{\rho_{ref,a}}{\rho_{ref,0}}\right)\cdot {\left(\frac{\phi_{0}}{\phi_{a}}\right)}^{n_{a}},$$

For the DI water case, the damage index was calculated based on Equation (13). The electrical conductivity of pore solution was considered not to change before and after the FTCs. Because the damage index was applied to quantify the degree of mechanical degradation, the 1-damage index was considered as the remaining mechanical property and compared with the normalized flexural strength and elastic modulus. As shown in Figure 12a, the average ratio of the 1-damage index to the normalized elastic modulus is 1.093, with coefficient of variation of 4.27%. Clearly, the mechanical degradation evaluation based on electrical measurements has an acceptable agreement with the results from the three-point bending tests. Although the results of severely-damaged sample cases are scattered, the results are still considered as acceptable. Because it is believed that the mechanical property measurements may be affected slightly during the three-point bending test preparation, as the samples were rather fragile, the comparison preliminarily verified the damage evaluation model based on electrical measurements. Therefore, after careful examination, it is concluded that the damage index can be used to evaluate the damage. Although it is true that there is difficulty to utilize the electrical measurement on site, it can be used in practice in the future and in the meantime it is useful for laboratory testing. In addition, for the specimens saturated with NaCl solutions, the data of electrical conductivity of pore solution before and after FTCs are lacking. To evaluate the salt-frost damage of concrete confidently, further experimental investigation is still required for verification.

## 5. Conclusions

In this paper, the electrical and mechanical responses of mortar at the meso-scale saturated with DI water and 5 and 15 wt.% NaCl solutions under FTCs were experimentally studied. Different electrical parameters were examined for evaluation of the salt-frost damage of mortar specimens. From the results and discussions, the following conclusions could be reached:

(1) Based on the examination of the relationship between electrical characteristics and mechanical property degradation, a damage evaluation model was developed and the electrical resistivity at reference temperature was the input. An acceptable agreement was reached between the proposed damage index from electrical analysis and the change in mechanical properties obtained from three-point bending tests, which preliminarily verified the reliability of the developed model.
(2) In the sealed condition under the FTCs, the specimens saturated with NaCl solutions could be severely damaged. The electrical resistivity values at both the highest and lowest temperatures change with the damage development. However, they show different tendency compared with previous studies due to the existing NaCl and smaller specimen dimension. The reduction in the electrical resistivity at the lowest temperature was not consistent with that of the elastic modulus and the flexural strength, due to the contradictory effects of connectivity and ice content increase, thus cannot be an efficient index for frost damage evaluation.
(3) The activation energy of mortar is not steady with frost damage development and the variation is insignificant, which is different from the observations from specimens with large size in a previous study [35]. Since a clear reduction tendency was not observed from the test results, it may not be an appropriate index for mechanical property-degradation evaluation.
(4) From the absorption test results, after the FTCs, there was insignificant change in the porosity, but the connectivity clearly increased. It reveals that severe frost damage can affect the pore size distribution, but does not necessarily have a large influence on the total porosity.
(5) Freezing and thawing points were detected from the relationship between the electrical resistivity and temperature. From the previous report, the freezing point increases with FTCs. However, in our meso-scale test results such a trend was not observed. The frost damage degree cannot simply be evaluated based on freezing and thawing points.

## Figures and Tables

**Table 1:** Mix Proportions and Properties of Mortar [37].

Water: Cement Ratio (W/C)	Water kg/m^3^	Cement kg/m^3^	Fine Aggregate kg/m^3^	Bulk dry Density (*ρ_b_*) kg/m^3^	Skeletal Density (*ρ*_0_) kg/m^3^	Porosity
0.3	292	974	1066	2173	2676	0.188
0.5	292	584	1397	2137	2688	0.205
0.7	292	418	1538	2125	2703	0.214

**Table 2:** Electrical resistivity values at the reference temperature (23 °C) and the lowest temperature (−28 °C) at the first cycle. Electrical resistivity at temperature 23 °C (Ω m).

	Electrical Resistivity at Temperature 23 °C (Ω m)				Electrical Resistivity at Temperature −28 °C (Ω m)		
	W/C = 0.3		W/C = 0.5	W/C = 0.7	W/C = 0.3	W/C = 0.5	W/C = 0.7
DI water		129.6	112.1	95.2	4622.5	3986.5	4745.5
5 wt.% NaCl		30.2	17.2	13.0	10,836.0	5500.6	1879.0
15 wt. % NaCl		11.0	11.6	6.2	762.2	476.9	296.4
